# Enhancement of productive performance, nutrient digestibility, and meat quality in broilers subjected to cyclic heat stress via different organic and inorganic chromium concentrations

**DOI:** 10.5194/aab-69-251-2026

**Published:** 2026-04-28

**Authors:** Youssef Abdelwahab Attia, Nicola Francesco Addeo, Fulvia Bovera, Rashed Abdullah Alhotan, Khalid Ali Asiry, Mohamed Alsaeed Al-Banoby, El-Shahat Mohamed Qota, Adel Daifallah Al-qurashi, Ahmed Shaban Awad

**Affiliations:** 1 Sustainable Agriculture Research Group, Agriculture Department, Faculty of Environmental Sciences, King Abdulaziz University, Jeddah 21589, Saudi Arabia; 2 Sustainable Agriculture Research Group, Department of Veterinary Medicine and Animal Production, University of Napoli Federico II, via F. Delpino,1, 80137, Napoli, Italy; 3 Department of Animal Production, College of Food and Agricultural Sciences, King Saud University, Riyadh 11451, Saudi Arabia; 4 Al-Shamel Animal Feed Factory, Industrial Area, Hail 55411, Saudi Arabia; 5 Department of Poultry Nutrition, Animal Production Research Institute, Agriculture Research Center, Ministry of Agriculture, Dokki, Giza, Egypt; 6 Rabbits, Turkey and waterfowl Department, Animal Production Research Institute, Agriculture Research Center, Ministry of Agriculture, Dokki, Giza, Egypt

## Abstract

Dietary chromium (Cr) supplementation of chickens may be a tool to reduce heat stress and its associated consequences. This study aims to investigate the effect of supplementation with various levels of inorganic and organic Cr on productive performance, nutrient digestibility, and meat quality in broiler chickens subjected to cyclic heat stress. A total of 245 as-hatched, 1 d old broiler chickens were randomly assigned to seven treatments, each with seven replicates of five birds. The control group received a basal diet without Cr supplementation. In the six other groups, chickens were fed a basal diet supplemented with 100, 200, and 400 ppb of organic and inorganic Cr. From days 25 to 42 d of age, the birds were subjected to heat stress for 3 consecutive days per week. Within chromium-supplemented treatments, the interaction between source and level of Cr influenced body weight gain (BWG) and feed conversion ratio (FCR) ( P<0.01 ), with the best values obtained at 400 ppb of organic Cr and 100 ppb of inorganic Cr. Dietary supplementation with organic Cr resulted in higher apparent digestibility (AD) of organic matter (OM; P<0.05 ) and crude protein (CP; P<0.01 ), while Cr levels ( P<0.01 ) affected AD of OM, CP, and ether extract (EE), with the best values observed at 200 and 400 ppb of Cr. Supplementation with 400 ppb of organic Cr or 100 ppb of inorganic Cr improved the growth performance and nutrient digestibility of broiler chickens raised under heat stress conditions. These findings align with the objective of the study and support the use of source-specific chromium supplementation strategies to mitigate cyclic heat stress in broilers.

## 1 Introduction

The adverse impacts of high ambient temperatures on poultry survival, performance, and product quality are extensively documented, presenting ongoing economic challenges for many agricultural enterprises. Heat-stressed poultry exhibit reduced feed intake, accompanied by changes in body composition characterized by heightened lipid deposition and diminished protein content in the muscle (Abdel-Moneim et al., 2021). Thermal stress induces high production of free radicals, compromising the meat quality and animal health, alongside acidosis, which reduces meat water-holding capacity, impairing its texture (Nawaz et al., 2021) and disrupting nutrient transporter expression (Orhan et al., 2019). Effective management practices involving the maintenance of appropriate temperature and humidity in modern poultry farms can mitigate heat stress (Saleh et al., 2023). However, such approaches are costly, increasing production expenses, and remain inaccessible for many regions in developing countries (Nawab et al., 2018). Chromium (Cr) supplementation in animal diets is gaining importance in livestock farming as a heat stress alleviator (Shan et al., 2020; Wang et al., 2023; Dalólio et al., 2024; Apalowo et al., 2024). Cr supplementation at 1 mg kg^−1^ or at 0.687 mg kg^−1^ has been recommended to mitigate the detrimental effects of heat stress in poultry by Piray and Foroutanifar (2021) and Kim et al. (2023), respectively, irrespective of the Cr source. Cr can be administered in diets as either inorganic or organic forms. Four forms of organic Cr are commercially available: Cr propionate, Cr picolinate, Cr methionine, and Cr yeast. Chromium picolinate, approved by the FDA for veterinary use in 1996, was introduced first (Chandrasekar and Balakrishnan, 2019). Cr plays a crucial role in carbohydrates, protein, and lipid metabolism through insulin signalling, enhancing amino acid and glucose absorption in poultry skeletal muscles (Chandrasekar and Balakrishnan, 2019). Additionally, Cr activates insulin receptors and mobilizes glucose transporter type 4 (GLUT 4) to improve glucose uptake (Vincent, 2015), potentially enhancing nutrient utilization and production performance. However, the effects of Cr supplementation on broiler chicken production remain contentious. While some studies indicate potential improvements in productivity and carcass characteristics (Huang et al., 2016; Lu et al., 2019; Hayat et al., 2020; Dalólio et al., 2021; Youssef et al., 2022; Fraz et al., 2023), others report no significant effects (Lee et al., 2003; Souza et al., 2010; Kim et al., 2021). Orhan et al. (2019) showed improved nutrient digestibility in hens supplemented with chromium picolinate or chromium histidinate, underscoring the trace element's importance in poultry nutrition through increased nutrient transporter expression. The regulatory status of chromium (Cr) as a feed additive varies across different geographical regions. In the European Union, chromium is not authorized as a general nutritional additive; however, specific chromium compounds may be permitted only following a scientific assessment by the European Food Safety Authority (EFSA) and formal authorization under regulation (EC) no. 1831/2003 for defined animal species and inclusion levels. In the United States, chromium propionate is approved by the Food and Drug Administration (FDA) as a feed additive for food-producing animals, including poultry, under specific conditions of use and maximum inclusion limits. In Saudi Arabia, the use of chromium-containing feed additives is subject to product-specific registration and approval by the relevant competent authorities, and no general authorization for chromium as a feed additive is publicly available. This study aims to compare the efficacy of organic and inorganic chromium at various concentrations in alleviating the effects of heat stress on broiler chickens, providing a comprehensive study of their impact on growing performance. Additionally, the research contributes to identifying the optimal dosages of both organic and inorganic chromium to maximize productivity and nutrient digestibility in broilers exposed to heat stress. Finally, this research contributes to existing literature on different chromium sources, offering detailed insights into their effects on specific nutrient digestibility and enhancing nutritional management of heat-stressed poultry.

**Table 1:** Ingredients and chemical composition of experimental diets.

Ingredients (g kg^−1^)	Starter phase	Grower phase	Finisher phase
	(1–14 d of age)	(15–30 d of age)	(31–42 d of age)
Yellow corn	550.0	617.9	682.0
Soybean meal	324.0	260.0	212.0
Corn gluten meal	55.0	54.0	40.0
Limestone	11.0	11.2	11.0
Dicalcium phosphate	17.5	15.0	13.0
Vitamin and mineral premix^1^	3.0	3.0	3.0
NaCl	3.0	3.0	3.0
DL-methionine	3.1	3.0	2.0
L-lysine (HCL)	3.4	2.9	3.5
Vegetable oils	30.0	30.0	30.5
Total	1000	1000	1000
Analysed^2^ and calculated^3^ values			
Dry matter^2^, g kg^−1^	868.0	874.0	869.0
Metabolizable energy^3^, MJ kg^−1^	12.69	13.03	13.28
Crude protein^2^, g kg^−1^	221.0	193.4	178.0
Methionine^3^, g kg^−1^	6.9	6.0	5.1
Sulfur amino acids (SAAs)^3^, g kg^−1^	10.6	9.4	8.2
Lysine^3^, g kg^−1^	13.4	11.8	10.6
SAA / lysine ratio, %	79.0	80.0	77.0
Calcium^3^, g kg^−1^	9.1	8.5	7.9
Available phosphorus^3^, g kg^−1^	4.6	4.1	3.6
Crude fat^2^, g kg^−1^	52.1	61.3	71.9
Crude fibre^2^, g kg^−1^	47.0	43.3	47.5
Ash^2^, g kg^−1^	52.5	55.3	57.4
Chromium^2^, mg kg^−1^ diet	0.626	0.673	0.704

^1^ Per kilogram of diet: vitamin A, 24 mg; vitamin E, 20 mg; menadione, 2.3 mg; vitamin D3, 0.05 mg; riboflavin, 5.5 mg; calcium pantothenate, 12 mg; nicotinic acid, 50 mg; choline chloride, 600 mg; vitamin B12, 10 µ g; vitamin B6, 3 mg; thiamine, 3 mg; folic acid, 1 mg; d-biotin, 0.05 mg; Mn, 80 mg kg^−1^; Zn, 60 mg kg^−1^; Fe, 35 mg kg^−1^; Cu, 8 mg kg^−1^; Se, 0.60 mg kg^−1^. ^2^ Analysed values: dry matter, crude protein, crude fat (ether extract), crude fibre, and ash were determined according to AOAC (1995). Chromium content of the diets was determined by ICP-OES (Varian 720-ES) as described by Olajire and Ayodele (1997); chromium distribution homogeneity was verified by ICP-OES analysis of the final diets. ^3^ Calculated values: metabolizable energy, amino acids (methionine, sulfur amino acids, and lysine), calcium, and available phosphorus were calculated based on ingredient composition and NRC (1994) nutrient tables/recommendations.

## 2 Materials and methods

A total of 245 Cobb500 broiler chicks (1 d old, both sexes) were wing-banded and randomly distributed based on similar initial body weight in a completely randomized design with seven treatments, each including seven replicates of five chicks. The sample size was chosen for logistical management reasons while maintaining an adequate number of replicates to obtain statistical power and minimize the effect of chance on the results. The choice of seven treatments reflects the desire to examine both the response to the presence or absence of chromium and the effects of specific concentrations of each source as efficiently as possible. The inclusion of a chromium-unsupplemented control group allows for a clear comparison with the organic and inorganic chromium treatments while maintaining the power of the experiment with a simple design. This approach allows us to isolate the main effect of different chromium concentrations and answer our hypothesis. Each replicate was maintained in a battery brooder (metal cage) with a size of 35 cm × 25 cm × 30 cm (L × W × H). The negative control group was fed a basal diet without Cr supplementation. In the six other groups, chicks were fed the same basal diet supplemented with 100, 200, and 400 ppb of organic (Cr picolinate, C18H12N3O6Cr; Nowfoods.com, made in Canada and quality tested in Bloomingdale, IL, USA) and inorganic Cr (Cr chloride, CrCl3; Muby Chemicals of the Mubychem Group, Mubychem, western India). The Cr content of the diets was determined using a Varian 720-ES ICP–optical emission spectrometer (ICP-OES), as described by Olajire and Ayodele (1997). The level of both organic and inorganic chromium was chosen based on a review of the scientific literature and the indications of the producers. Previous studies indicate not only that organic chromium, such as picolinate, tends to have a higher absorbability under standard conditions, but also that inorganic chromium chloride can positively affect growth performance at optimal levels under stress conditions (Jain et al., 2018; Arif et al., 2019). The responses to chromium supplements may vary greatly depending on the environmental context and the specific needs of each organism under stress. The ingredients and chemical composition of the basal starter, grower, and finisher diets are presented in Table 1. Chromium was mixed directly into the chicken diet at the concentrations indicated. Chromium sources were first premixed using a blender with a small amount of the basal diet to obtain a homogeneous micro-premix, which was then incorporated into the complete mash diet using mechanical mixing. The homogeneity of chromium distribution was verified by ICP-OES analysis of the final diets. Although pelleting can further improve homogeneity, mash diets are commonly used in experimental trials to avoid heat-induced degradation of organic chromium forms. During 1–24 d of age, the birds were kept under the same temperature conditions (32, 30, and 28 °C during the first, second, and third week, respectively). The chickens were housed in a semi-open room. Then, during 25–42 d of age, birds were kept for 3 successive days (Monday, Tuesday, and Wednesday) weekly at 36 ± 2 °C and 75 %–85 % relative humidity from 10:00 to 17:00. All experimental groups, including the control group, were exposed to the same heat stress conditions; therefore, the control birds represent heat-stressed broilers fed a basal diet without chromium supplementation. When not exposed to heat, the birds were kept under thermoneutral conditions (25 ± 2 °C). During the entire experimental period, the chicks were kept under similar managerial and hygienic conditions. Chickens were provided with a 23 : 1 daily light : dark cycle during the experimental period. Chickens were fed corn–soybean meal mash diets during days 1–14 d (starter diet), 15–30 d (grower diet), and 31–42 d (finisher diet) of age. The ingredient and chemical composition of the experimental diets are reported in Table 1.

**Table 2:** LS means of productive performance of broilers at 42 d of age fed diets with different sources and concentrations of chromium supplementation ( n=7 replicates per treatment).

Treatments		BWG (g)	FI (g)	FCR (kg kg^−1^)	EPEI
Chromium source					
Organic		1865^a^	3046	1.64	250
Inorganic		1830^b^	3030	1.67	252
P value		0.03	0.490	0.068	0.791
Chromium concentrations (ppb)					
0		1614^c^	3035	1.87 ^a^	174
100		1869^a^	3036	1.64 ^b^	258
200		1788^b^	3024	1.69 ^b^	238
400		1884^a^	3054	1.63 ^b^	257
P value		0.001	0.182	0.039	0.128
Interaction (sources × concentrations of chromium)					
Control	0	1614^c^	3035	1.87^a^	173
Organic	100	1835^b^	3052	1.67^bc^	265
	200	1807^b^	3029	1.67^bc^	242
	400	1954^a^	3058	1.57^d^	244
Inorganic	100	1905^a^	3038	1.61^cd^	251
	200	1770^b^	3021	1.71^b^	235
	400	1817^b^	3050	1.68^bc^	270
SEM		20.1	27.2	0.026	15.3
P value		0.0001	0.089	0.01	0.148

^a,b,c^ Means in a column under similar treatment conditions not sharing the same superscript are significantly different ( P<0.05 ). SEM – pooled standard error of the mean. BWG – body weight gain. FI – feed intake. FCR – feed conversion ratio. EPEI – European Production Efficiency Index. The control (0 ppb supplemented Cr) is shown for comparison; the source × level interaction refers to chromium-supplemented treatments only (100–400 ppb). The 0 ppb supplemented Cr control was compared with the chromium-supplemented treatments by planned contrasts within the GLM analysis.

**Table 3:** LS means of apparent digestibility (AD) of nutrients in broilers at 42 d of age fed diets with different sources and concentrations of chromium supplementation ( n=5 broilers (replicates) per treatment).

Treatments		AD of nutrients, %					
		DM	OM	CP	EE	CF	Ash
Chromium source							
Organic		76.7	83.7^a^	86.1^a^	82.2	13.91	21.93
Inorganic		76.7	80.0^b^	83.2	78.5	15.52	21.57
P value		0.28	0.03	0.01	0.60	0.84	0.84
Chromium concentration, ppb							
0		75.9	78.4^b^	83.2^b^	77.3^b^	15.13	21.23
100		75.8	80.4^ab^	83.4^ab^	79.4^ab^	14.46	21.82
200		76.9	82.2^a^	84.2^a^	80.3^a^	14.38	21.64
400		77.4	83.1^a^	86.5^a^	81.3^a^	15.41	21.78
P value		0.51	0.01	0.01	0.02	0.06	0.39
Interaction (source × concentrations of chromium)							
Control	0	75.9	78.4	83.2	77.3	15.13	21.23
Organic	100	77.3	82.3	84.4	80.8	13.38	20.84
	200	76.4	83.4	85.8	82.3	13.65	22.45
	400	76.5	85.5	88.1	83.4	14.56	22.34
Inorganic	100	74.3	78.5	82.3	77.9	15.38	22.78
	200	77.4	80.9	82.6	78.3	14.94	20.68
	400	78.3	80.7	84.8	79.2	16.14	20.98
SEM		2.87	2.24	1.72	2.45	2.77	3.44
P value		0.38	0.42	0.68	0.43	0.91	0.57

^a,b^ Means in a column under similar treatment conditions not sharing the same superscript are significantly different at 5 %. SEM – pooled standard error of the mean. DM – dry matter, OM – organic matter, CP – crude protein, EE – ether extract, CF – crude fibre. The control (0 ppb supplemented Cr) is shown for comparison; the source × concentration interaction refers to chromium-supplemented treatments only (100–400 ppb).

Feed and water were provided ad libitum. Vaccination against avian influenza (H5N2) was performed under the advice of veterinary authorization. Chicks were vaccinated against Newcastle disease virus (NDV) using a live attenuated vaccine, Hitchner B1 (commercial name: ND Hitchner B1, MSD Animal Health, Intervet, the Netherlands), on day 7 of age, and the LaSota strain (commercial name: ND LaSota, MSD Animal Health, Intervet, the Netherlands) on days 20 and 30. Birds were vaccinated against avian influenza (H5N2) using an inactivated vaccine (commercial name: avian influenza H5N2, Zoetis, USA) on day 9 and against infectious bursal disease (Gumboro) using a live vaccine (commercial name: IBD Intermediate Plus, Ceva Santé Animale, France) on days 14 and 24. Birds and rations were weighed on the first and last (42) days of the experimental period to calculate body weight gain (BWG), feed intake (FI), feed conversion ratio (FCR), and mortality rate (MR). The European Production Efficiency Index (EPEI) was calculated as described by Metwally et al. (2020). At 42 d of age, five male chicks per treatment were randomly selected from the seven replicates and housed individually for the digestibility assay using the total excreta collection method. Chicks were fasted for 24 h and then fed their corresponding experimental diets for 72 h, in which feed intake and excreta produced during the period were determined. The excreta were collected for each replicate, cleaned of feathers and feed, weighed, and dried in a forced-air oven at 70 °C for 36 h. Samples were then ground and placed in screw-top glass jars until analysis of dry matter, organic matter, nitrogen, ether extract, and crude fibre (AOAC, 1995). The apparent digestibility (AD; %) of the nutrients was calculated using the following formula: [(IN - NE) / IN] × 100, where IN is the ingested nutrient (g), and NE is the nutrients in the excreta (g). Only for nitrogen was fecal N used instead of NE. The procedure described by Jakobsen et al. (1960) was used to separate fecal nitrogen from urine nitrogen in the excreta samples. At 42 d of age, eight broilers of both sexes were randomly selected from each treatment to cover all replicates (seven replicates), with one replicate contributing two birds. Sex was recorded, and birds were weighed after overnight fasting, slaughtered, and feather-picked. The total inedible parts (head, legs, and inedible viscera) were set aside, and the carcass was weighed. The internal organs, including the liver, gizzard, heart, spleen, pancreas, bursa of Fabricius, abdominal fat, and intestine, were separated and individually weighed; their relative weights were expressed as a percentage of the live body weight. The intestine weight was recorded in grams and expressed as a percentage of live body weight at slaughter, and the length (cm) of the intestine was measured as indicators of gastrointestinal development and potential adaptive responses to heat stress and dietary chromium supplementation. Eight samples per treatment, composed of 50 % breast meat and 50 % thigh meat, were weighed and kept in an electric drying oven at 70 °C for 24 h until a constant weight was achieved. The dried flesh was finely ground using a suitable mixer to pass through a sieve (1 mm) and then carefully mixed. The air-dried samples were stored in airtight glass containers for subsequent analyses. The dry matter, nitrogen, fat, and ash contents were determined according to AOAC (1995). The physical characteristics of meat (breast and thigh meat) were determined using eight fresh-meat samples per treatment. Water-holding capacity (WHC) and tenderness were measured according to the method of Volvoinskaia and Kelman (1962), in which 0.3 g minced meat tissues were put under an ashless filter paper and pressed for 10 min using 1 kg weight. Two zones formed on the filter paper. Surface areas were measured using a planimeter. WHC was calculated by subtracting the internal zone from the outer zone. The internal zone is due to meat pressing, which indicates tenderness. The pH value was measured using 10.0 g of prepared samples of meat and drip blended with 50 mL of distilled water for 10 min, and then the pH value was measured (Aitken et al., 1962). The colour intensity of the meat and drip was determined by shaking 10 g with 50 mL distilled water in a dark room for 10 min. The samples were filtered, and the colour intensity (absorbency) was measured photometrically at 543 nm (Husani et al., 1950). Mortality during the experiment was negligible and did not significantly affect the final data. Animals were carefully monitored and kept in suitable conditions to minimize the impact of thermal stress on mortality.

**Table 4:** LS means of percentage of carcass and abdominal fat of broilers at 42 d of age fed diets with different sources and concentrations of chromium supplementation ( n=8 broilers (replicates) per treatment.

Treatments		Percentage of carcass and abdominal fat, %			
		Dressing	Front part	Hind part	Abdominal fat
Chromium source					
Organic		68.9	34.6	29.3	0.808
Inorganic		69.2	33.9	30.5	0.670
P value		0.820	0.343	0.081	0.324
Chromium concentrations, ppb					
0		69.7	35.2	30.4	0.50
100		68.8	35.0	29.8	0.67
200		69.4	34.3	30.5	0.82
400		68.9	33.4	29.4	0.73
P value		0.902	0.255	0.386	0.675
Interaction (sources × concentrations of chromium)					
Control	0	69.7	35.2	30.4	0.501
Organic	100	70.2	36.0	29.4	0.669
	200	69.3	34.4	30.3	1.079
	400	67.2	33.4	28.4	0.677
Inorganic	100	67.3	34.0	30.2	0.663
	200	69.3	34.2	30.7	0.556
	400	70.6	33.4	30.5	0.792
SEM		1.540	0.970	0.764	0.170
P value		0.139	0.543	0.514	0.147

SEM – pooled standard error of the mean. Values are least-squares means averaged over sex; sex and its interactions were included in the statistical model but are not shown because they were not statistically significant ( P>0.05 ). The control (0 ppb supplemented Cr) is shown for comparison; the source × concentration interaction refers to chromium-supplemented treatments only (100–400 ppb).

### Statistical analysis

Data were analysed using the GLM procedure (PROC GLM) of SAS software (SAS Institute Inc., Cary, NC, USA). The experiment was conducted as a completely randomized design with seven dietary treatments and seven replicates (cages) per treatment, with five birds per replicate. The dietary treatments consisted of an unsupplemented control diet and six chromium-supplemented diets arranged as two chromium sources (organic or inorganic) at three supplemental levels (100, 200, or 400 ppb). Because the control diet did not include a chromium source, the source × level interaction was evaluated only among chromium-supplemented treatments (100–400 ppb), whereas the 0 ppb unsupplemented control group was retained as an external reference for comparison. Planned contrasts were additionally used to compare the unsupplemented control with the chromium-supplemented source × level combinations. Growth performance variables (body weight gain, feed intake, feed conversion ratio, and European Production Efficiency Index) were analysed using replicate (cage battery brooder or metal cage) as the experimental unit according to the following model:

Y=μ+CRS+CRLV+(CRS×CRLV)+e,

where Y is the observed value of the response variable for each replicate, μ is the overall mean, CRS is chromium source, CRLV is chromium level, and e is the residual error. For carcass traits, internal organs, and the chemical and physical traits of meat, individual bird measurements obtained at 42 d of age were analysed according to the following model:

1 $$\begin{array}{rl} Y & =\mu +\mathrm{CRS}+\mathrm{CRLV}+\mathrm{Sex}+(\mathrm{CRS}\times \mathrm{CRLV})\\ & +(\mathrm{CRS}\times \mathrm{Sex})+(\mathrm{CRLV}\times \mathrm{Sex})\\ & +(\mathrm{CRS}\times \mathrm{CRLV}\times \mathrm{Sex})+e, \end{array}$$

where Y is the observed value of the response variable for each individual bird, μ is the overall mean, Sex is the effect of sex, and e is the residual error. Because the experimental design was not fully factorial owing to the presence of the 0 ppb unsupplemented control group, least-squares means were used. Results are presented as least-squares means for chromium source, chromium level, and their interaction within chromium-supplemented treatments only (100–400 ppb), whereas the 0 ppb control group is shown for comparison. Sex effects were retained in the model and are mentioned only when statistically significant; otherwise, results are presented averaged over sex, consistent with the layout of Tables 4–7. Normality and homogeneity of variances were assessed using the Shapiro–Wilk test (Shapiro and Wilk, 1965) and Levene's test (Levene, 1960), respectively. Percentage data were log10-transformed when needed to improve normality. Mean differences were separated using the Student–Newman–Keuls test. Mortality rate was analysed using the chi-square test. Differences were considered significant at P<0.05 .

## 3 Results

### Productive performance

There was no significant effect of Cr source on FI, FCR, and EPEI or of Cr levels on FI and EPEI. Within the chromium-supplemented treatments (organic vs. inorganic at 100–400 ppb), the source × level interaction affected BWG ( P<0.0001 ) and FCR ( P<0.01 ) (Table 2).

Considering organic Cr, the BWG of birds receiving 400 ppb was higher than that of the groups receiving 100 and 200 ppb. Conversely, when birds were fed inorganic Cr, a better BWG was observed in the group receiving 100 ppb of Cr. Broilers fed diets containing Cr had a greater BWG than those in the control group. There was no difference between the BWG of birds receiving 400 ppb of organic Cr and those receiving diets with 100 ppb of inorganic Cr. The best FCR values were obtained with dietary supplementation with 400 ppb of organic Cr and 100 ppb of inorganic Cr. As FI was not affected, FCR reflected the improvement in the BWG. Supplementation of diets with organic Cr resulted in higher apparent digestibility (AD) of organic matter (OM; P<0.03 ) and crude protein (CP; P<0.01 ), whereas Cr levels affected ( P<0.01 ) the AD of OM, CP, and EE (Table 3), with the best values obtained with 200 and 400 ppb of Cr.

Neither carcass characteristics nor body organs were influenced ( P>0.05 ) by the source × level interaction when averaged over sex; however, broilers fed diets containing organic Cr had a lower heart percentage ( P<0.005 ) (Tables 4 and 5). Sex and its interactions with chromium source and level were tested for slaughter traits, but no significant effects were detected; therefore, results are presented averaged over sex.

**Table 5:** LS means of internal organs in broilers at 42 d of age fed diets containing different supplementations chromium sources and concentrations ( n=8 broilers (replicates) per treatment).

Treatments		Internal organs, %						
		Heart	Pancreas	Gizzard	Liver	Proventriculus	Intestine	
							Weight, %	Length, cm
Chromium source								
Organic		0.503^b^	0.223	1.73	2.31	0.381	3.76	151
Inorganic		0.582^a^	0.214	1.73	2.14	0.386	3.96	162
P value		0.005	0.482	0.994	0.090	0.829	0.195	0.003
Chromium concentrations, ppb								
0		0.528	0.249	1.71	2.48	0.430	4.63	157
100		0.533	0.217	1.69	2.37	0.376	3.97	157
200		0.545	0.229	1.82	2.21	0.399	3.80	155
400		0.549	0.209	1.69	2.11	0.376	3.81	158
P value		0.865	0.467	0.186	0.129	0.609	0.602	0.765
Interaction (sources × concentrations of chromium)								
Control	0	0.528	0.249	1.71	2.48	0.430	4.63	157
Organic	100	0.497	0.228	1.71	2.56	0.371	3.83	154
	200	0.475	0.241	1.78	2.29	0.396	3.89	149
	400	0.536	0.201	1.70	2.10	0.378	3.57	151
Inorganic	100	0.568	0.207	1.66	2.17	0.381	4.10	160
	200	0.615	0.217	1.86	2.14	0.404	3.71	162
	400	0.563	0.218	1.67	2.11	0.373	4.05	166
SEM		0.033	0.016	0.082	0.126	0.027	0.181	4.312
P value		0.240	0.367	0.688	0.286	0.954	0.188	0.501

^a,b^ Means in a column under similar treatment conditions not sharing the same superscript are significantly different at 5 %. SEM – pooled standard error of the mean. Sex effect and sex × chromium treatment interactions were tested but are not shown. Values are least-squares means averaged over sex; sex and its interactions were included in the statistical model but are not shown because they were not statistically significant ( P>0.05 ). The control (0 ppb supplemented Cr) is shown for comparison; the source × concentration interaction refers to chromium-supplemented treatments only (100–400 ppb).

The treatments did not affect ( P>0.05 ) the chemical composition, physical characteristics, or meat quality of broilers (Tables 6 and 7).

**Table 6:** LS means of meat chemical composition of 42 d old broiler chickens fed diets with different supplementations chromium sources and concentrations, on a fresh-weight basis ( n=8 broilers (replicates) per treatment).

Treatments		Chemical composition, %			
		Dry matter	Protein	Lipids	Ash
Chromium source					
Organic		25.0	18.9	4.93	1.20
Inorganic		25.0	18.8	5.00	1.19
P value		0.942	0.586	0.422	0.683
Chromium concentrations, ppb					
0		25.1	18.9	5.01	1.18
100		25.0	18.8	4.99	1.19
200		25.0	18.9	4.89	1.19
400		25.0	18.7	5.04	1.20
P value		0.996	0.532	0.414	0.606
Interaction (sources × concentrations of chromium)					
Control	0	25.1	18.9	5.01	1.18
Organic	100	25.1	18.8	4.98	1.19
	200	25.0	19.0	4.80	1.19
	400	25.0	18.8	5.01	1.21
Inorganic	100	25.0	18.8	5.00	1.19
	200	25.1	18.9	4.97	1.18
	400	25.1	18.7	5.05	1.19
SEM		0.095	0.135	0.087	0.011
P value		0.939	0.951	0.774	0.880

SEM – pooled standard error of the mean. Values are least-squares means averaged over sex; for the traits reported in this table, sex and its interactions were included in the statistical model but are not shown because they were not statistically significant ( P>0.05 ). The control (0 ppb supplemented Cr) is shown for comparison; the source × concentration interaction refers to chromium-supplemented treatments only (100–400 ppb).

**Table 7:** LS means of physical characteristics of meat from broiler chickens at 42 d of age fed diets containing different supplementations chromium sources and concentrations ( n=8 broilers (replicates) per treatment).

	Physical characteristics of meat				
Treatments		pH	Colour, absorbance	Tenderness,	WHC,
			at 543 nm	cm^2^/0.3 g of meat	cm^2^/0.3 g of meat
Chromium source					
Organic		6.66	0.196	2.84	4.90
Inorganic		6.65	0.190	2.84	4.85
P value		0.711	0.386	0.966	0.410
Chromium concentrations, ppb					
0		6.73	0.206	2.84	4.85
100		6.69	0.196	2.83	4.88
200		6.67	0.192	2.85	4.85
400		6.63	0.191	2.84	4.85
P value		0.381	0.812	0.948	0.881
Interaction (sources × concentrations of chromium)					
Control	0	6.73	0.206	2.84	4.85
Organic	100	6.69	0.199	2.83	4.91
	200	6.68	0.196	2.83	4.91
	400	6.63	0.192	2.86	4.89
Inorganic	100	6.64	0.193	2.83	4.80
	200	6.70	0.187	2.87	4.87
	400	6.63	0.191	2.83	4.88
SEM		0.044	0.008	0.070	0.079
P value		0.765	0.808	0.896	0.799

SEM – pooled standard error of the mean. WHC – water-holding capacity. Values are least-squares means averaged over sex; for the traits reported in this table, sex and its interactions were included in the statistical model but are not shown because they were not statistically significant ( P>0.05 ). The control (0 ppb supplemented Cr) is shown for comparison; the source × concentration interaction refers to chromium-supplemented treatments only (100–400 ppb).

## 4 Discussion

The calculated Cr content of the experimental diets ranged from 0.626 to 0.704 mg kg^−1^ diet. Although the basal diets contained measurable background chromium, native Cr in conventional feed ingredients is generally characterized by low availability, which may help explain the response observed even at the lowest supplemental level. The Cr content in corn, soybean meal, and corn gluten meal was 0.055, 0.124, and 0.9 ppm, respectively. The observed Cr content in the feed ingredients used in our study, specifically 0.055 ppm in corn and 0.124 ppm in soybean meals, is indeed lower than the values reported by Bohlmann (2012), who identified Cr concentrations of 0.9 ppm in corn and 0.2 ppm in soybean meal. This discrepancy could be attributed to variations in agricultural practices, soil composition, and environmental factors that influence the mineral content of crops. Moreover, the differences in the Cr content could also result from methodological variations in measuring Cr concentrations or the specific batches of ingredients used in the study. There are no established Cr requirements for poultry, emphasizing the importance of studying the impact of different Cr sources and levels on broiler performance. Cr has been studied as an anti-heat-stress agent for broilers (Dalólio et al., 2021; Kim et al., 2021; Piray and Foroutanifar, 2021; Kim et al., 2023). The markedly lower body weight gain and poorer feed conversion ratio observed in the control group confirm the effectiveness of the heat stress model applied in this study and are consistent with the well-documented negative effects of cyclic heat stress on broiler performance. Because no thermoneutral group was included, the control group was intentionally designed to serve as a heat-stressed reference, allowing the effects of chromium supplementation to be evaluated specifically under thermal stress conditions. Although birds were exposed to high ambient temperatures for 3 consecutive days per week rather than continuously, the repeated cyclic exposure (36 ± 2 °C for 7 h d^−1^ from 25 to 42 d of age) can be classified as chronic cyclic heat stress. This experimental model is widely used in poultry research to simulate field conditions in which broilers are subjected to recurrent heat load over extended periods. The absence of a thermoneutral control group represents a limitation of the present study, as it does not allow a direct comparison between thermoneutral and heat-stressed conditions. However, the experimental design was specifically aimed at evaluating the relative effects of organic and inorganic chromium supplementation under heat stress conditions. Therefore, the control group consisted of heat-stressed birds fed a basal diet without chromium supplementation and served as an appropriate reference to assess chromium-mediated responses under identical environmental conditions. Heat stress triggers the release of corticosteroids, affecting glucose and mineral metabolism and immune function (Siegel, 1995). It also increases Cr mobilization from tissues and excretion through urine (Sahin et al., 2002), potentially leading to marginal Cr deficiency or an increased Cr requirement (Feng et al., 2021). Supplementing broiler diets with Cr can reduce glucocorticoid levels, as reported by Bahrami et al. (2012). Organic Cr is absorbed more efficiently (25 %–30 %) than inorganic Cr (1 %–3 %) (Piva et al., 2003; Król et al., 2017). Moreover, the absorption rate is inversely proportional to the dietary Cr level (Kobla and Volpe, 2000; Vincent, 2000). Cr enhances insulin sensitivity, potentially stimulating protein synthesis and inhibiting proteolysis (Tesseraud et al., 2007). These show the differences in Cr utilization among sources and levels and warrant further research. The highest BWG and FCR values were observed in birds fed diets with 400 ppb of organic Cr and 100 ppb of inorganic Cr, with BWG surpassing that of the control group. Although organic chromium is four times more concentrated than inorganic chromium, its absorption by the body is very low. However, organic chromium improves nutrient absorption and protein deposition more effectively than inorganic chromium, which explains why better body weight gains (BWGs) are achieved at a concentration of 400 ppb, despite the low absorption rate. Increasing inorganic Cr levels worsened BWG and FCR, likely due to decreased digestibility rates. Arif et al. (2019) found that dietary Cr propionate at 400 ppb improved BWG, while FI and FCR were better at 200 ppb. Including Cr propionate (0.1 to 0.3 mg kg^−1^) in broiler diets improved BWG and FCR without affecting FI (Hayat et al., 2020). However, Ghazi et al. (2012) reported no effect of 0.6 and 1.2 mg kg^−1^ of Cr on broilers' performance under heat stress. Dalólio et al. (2021) also found no effect of Cr methionine (0.1–1.2 mg kg^−1^) on broilers' performance at 43 d of age. Wang et al. (2022) noted that 0.4 and 0.8 mg kg^−1^ Cr picolinate in heat-stressed broiler diets decreased BWG and FI and increased FCR. Organic Cr improved OM and CP digestibility more than inorganic Cr due to its higher absorption rate, affecting nutrient transporter expression. Cr supplementation alleviates the negative effect of heat stress on digestibility by increasing the expression of nutrient transporters in laying hens (Orhan et al., 2019). Diets with 200 and 400 ppb Cr resulted in better AD of OM, CP, and EE, enhancing nutrient digestibility. Hens fed diets with 1.6 ppm CrPic (12.43 % Cr) or 0.788 ppm CrHis (25.22 % Cr) per kg, providing 200 mg elemental Cr per tonne, showed better digestibility of dry matter, organic matter, and crude protein due to increased transporter expression for carbohydrates, fats, and amino acids in laying hens (Orhan et al., 2019). Wang et al. (2022) also found increased glucose transporter expression in the jejunum with 0.4 mg kg^−1^ Cr picolinate of heat-stressed broiler chickens. Broilers fed organic Cr had lower relative heart weights than those fed inorganic Cr. Heat stress reduces feed intake and circulating glucose (Pearce et al., 2013). Less absorbed inorganic Cr has a lower effect on insulin sensitivity, leading to less glucose uptake by myocardial cells. Energy metabolism shifts to fatty acid oxidation, forming advanced glycation end products that increase heart weight (Letonja and Petrovic, 2014). This study's findings align with those of other studies observing non-significant improvements in broiler carcass characteristics and internal organs (Bahrami et al., 2012; Ghanbari et al., 2012; Arif et al., 2019). Anandhi et al. (2006) reported reduced abdominal fat thickness and muscle cholesterol in Cr-supplemented broiler chickens. Cr supplementation increased protein content in broiler meat (Anandhi et al., 2006; Saikat et al., 2008; Toghyani et al., 2008). Organic Cr increased carcass weight and yields of liver, heart, spleen, and gizzard, while reducing abdominal fat in broilers (Sahin et al., 2003; Saikat et al., 2008; Noori et al., 2012). Dalólio et al. (2021) found a quadratic effect of Cr methionine (0.1–1.2 mg kg^−1^) on abdominal fat and the heart weight of broiler chickens, with optimal values at 0.75 and 0.43 mg kg^−1^ Cr, respectively. Hayat et al. (2020) also found increased heart weight with dietary Cr propionate (0.1–0.3 mg kg^−1^) in broiler chickens. Regarding meat quality, Souza et al. (2010) observed a quadratic effect of organic Cr (150–600 mg kg^−1^) on breast meat fat content in broilers, with a 7.03 % reduction at 218.2 mg kg^−1^ Cr. Untea et al. (2019) reported increased crude protein, Cr, Zn, and Fe levels in broiler meat with dietary Cr picolinate (0.2 and 0.4 mg kg^−1^). Other researchers have noted positive effects of Cr on meat quality of broilers (Huang et al., 2015). Potential environmental concerns related to chromium supplementation in animal nutrition should also be considered. Background chromium concentrations in agricultural soils typically range from 10–100 mg kg^−1^, and regulatory limits for total chromium in soils are generally set well above these values, depending on national and international guidelines. In the present study, chromium inclusion levels (100–400 ppb in feed) resulted in total dietary chromium concentrations far below levels commonly associated with environmental contamination. Furthermore, all chromium sources used were in the trivalent form (Cr^3+^), which is characterized by low mobility and limited bioavailability in soils compared with hexavalent chromium. Accordingly, when chromium is applied at nutritionally relevant levels and under controlled feeding conditions, the risk of environmental chromium accumulation through poultry manure is expected to be minimal. Nevertheless, long-term investigations evaluating chromium excretion and soil accumulation remain necessary to fully assess the environmental sustainability of chromium supplementation in poultry production systems.

## 5 Conclusions

In conclusion, supplementing broiler diets with 400 ppb of organic chromium (Cr) or 100 ppb of inorganic Cr from 1 to 42 d of age significantly enhances growth performance and nutrient digestibility under cyclic chromium inclusion levels underheat stress conditions. Specifically, organic Cr supplementation was found to improve feed digestibility more effectively compared to inorganic Cr at a higher concentration. These findings suggest that organic Cr is more beneficial in mitigating the negative effects of heat stress on broiler chickens. Furthermore, the study identifies optimal dosages for both organic (400 ppb) and inorganic (100 ppb) Cr that maximize production performance and nutrient utilization. This research provides valuable insights into the nutritional management of broiler chickens exposed to thermal stress, offering practical recommendations to enhance poultry health and efficiency. The results underscore the importance of selecting appropriate sources and dosages of Cr to improve the productivity and resilience of broiler chickens in challenging environmental conditions. However, concerns can be raised about the environmental impact of using chromium, both organic and inorganic, in chicken feed. Although the study highlights the benefits of Cr on performance and nutrient digestibility in chickens under heat stress, the accumulation of chromium in the environment could represent a potential risk, especially if the amounts used are not carefully managed. Future research could consider not only the long-term health effects on chickens, but also the environmental impact of using Cr in poultry production systems. Future studies should include thermoneutral control groups and long-term evaluations of chromium excretion and environmental accumulation to further strengthen conclusions regarding both physiological efficacy and environmental sustainability. In accordance with the primary objective of this research, the present results support the practical application of optimized chromium inclusion levels under cyclic heat stress conditions.

## Data Availability

The data that support the findings of this study are available upon formal request from the corresponding author and approval by the funding agent. The data are not publicly available due to privacy or ethical restrictions.
